# Bibliometric analysis of research on gut microbiota and bile acids: publication trends and research frontiers

**DOI:** 10.3389/fmicb.2024.1433910

**Published:** 2024-08-21

**Authors:** Xin Li, Can Lu, Xue Mao, Jiahong Fan, Jianting Yao, Jingjie Jiang, Lele Wu, Jingjing Ren, Jun Shen

**Affiliations:** ^1^Department of General Medicine and Geriatrics, Linping Campus, The Second Affiliated Hospital, Zhejiang University, Hangzhou, Zhejiang, China; ^2^Department of General Practice, The First Affiliated Hospital, Zhejiang University School of Medicine, Hangzhou, Zhejiang, China; ^3^Department of Colorectal Surgery and Oncology, Key Laboratory of Cancer Prevention and Intervention, Ministry of Education, The Second Affiliated Hospital of Zhejiang University School of Medicine, Hangzhou, Zhejiang, China; ^4^Department of Medical Oncology, The Second Affiliated Hospital of Zhejiang University School of Medicine, Hangzhou, Zhejiang, China; ^5^Cancer Institute, Key Laboratory of Cancer Prevention and Intervention, Ministry of Education, The Second Affiliated Hospital of Zhejiang University School of Medicine, Hangzhou, Zhejiang, China; ^6^Cancer Center, Zhejiang University, Hangzhou, Zhejiang, China; ^7^Department of Ultrasound in Medicine, The Second Affiliated Hospital of Zhejiang University School of Medicine, Zhejiang University, Hangzhou, China

**Keywords:** bile acid, gut microbiota, bibliometric, human, research hotspots

## Abstract

The gut microbiota is widely regarded as a “metabolic organ” that could generate myriad metabolites to regulate human metabolism. As the microbiota metabolites, bile acids (BAs) have recently been identified as the critical endocrine molecules that mediate the cross-talk between the host and intestinal microbiota. This study provided a comprehensive insight into the gut microbiota and BA research through bibliometric analysis from 2003 to 2022. The publications on this subject showed a dramatic upward trend. Although the USA and China have produced the most publications, the USA plays a dominant role in this expanding field. Specifically, the University of Copenhagen was the most productive institution. Key research hotspots are the gut–liver axis, short-chain fatty acids (SCFAs), cardiovascular disease (CVD), colorectal cancer (CRC), and the farnesoid x receptor (FXR). The molecular mechanisms and potential applications of the gut microbiota and BAs in cardiometabolic disorders and gastrointestinal cancers have significant potential for further research.

## 1 Introduction

The gut microbiota is a complex microbial ecosystem populated by approximately 100 trillion microbes harboring the human intestine, incorporating bacteria and other non-bacterial microorganisms (Lozupone et al., 2012). The principal bacteria phyla of the gut microbiota are *Bacteroidetes* and *Firmicutes*, along with *Proteobacteria* and *Actinobacteria* (Shanahan et al., 2021). Due to the application of new molecular techniques and advanced bioinformatics, it is recognized that the gut microbiota has a fundamental role in regulating host metabolism, immunity systems, and the central nervous system (Sonnenburg and Sonnenburg, 2019). Since intestinal microbiota contain approximately 10 million unique genes with the potential to conduct myriad more chemical reactions than humans (Li et al., 2014), bioactive compounds produced by these microbiomes are regarded as the primary mediators in the cross-talk of microbiota and host, including SCFAs, γ-aminobutyric acid (GABA), and branched-chain amino acids (BCAAs) (Mancin et al., 2023). Microbiota-derived bile acids (BAs) have recently been identified as significant signaling molecules involved in an array of host metabolisms (de Aguiar Vallim et al., 2013).

BAs comprise primary BAs synthesized in the hepatocytes and secondary BAs produced by the gut bacteria. Cholesterol 7α-hydroxylase is mainly responsible for the biosynthesis of the primary BAs cholic acid (CA) and chenodeoxycholic acid (CDCA) from cholesterol in the liver. After primary BAs are conjugated with glycine or taurine, they will be released into the duodenum to digest and absorb lipids and vitamins following the diet. Approximately 95% of BAs are reabsorbed into the portal vein via passive and active transport on the intestinal epithelium (Hofmann, 2009), and the remaining 5% may receive biochemical modifications by the distal gut microbiota (de Aguiar Vallim et al., 2013). The gut microbiota metabolizes the primary BAs into the secondary BAs primarily through deconjugation, dehydrogenation, and dihydroxylation (Sayin et al., 2013), which could reduce the toxicity of BAs and increase the diversity of the BA pool (Jones et al., 2008). Although deoxycholic acid (DCA) and lithocholic acid (LCA) are considered the major secondary BAs, a growing number of these BAs has been identified with the characterization of new bacterial enzymes and the advancement of BA detection methods (Collins et al., 2023), such as esterified BAs, isoDCA, ursodeoxycholic acid (UDCA), and amino acid-conjugated BAs.

Meanwhile, BA signaling activity dominantly depended on the activation of BA receptors, including nuclear receptors, such as the FXR and G protein-coupled receptors (TGR5) (Swann et al., 2011). Importantly, FXR is not only highly expressed but also intensively studied in the liver and ileum, while TGR5 has a broad expression in the gallbladder, placenta, lung, spleen, intestine, liver, brown and white adipose tissue, skeletal muscle, and bone marrow (Wahlström et al., 2016). Owing to the reabsorption of secondary BAs into the systemic circulation in the intestine, it can regulate various biological processes of the host via binding to the targeted receptors, including lipid and glucose metabolism, energy expenditure, hepatic bile synthesis, bacterial growth, and systemic inflammation (Swann et al., 2011; de Aguiar Vallim et al., 2013).

Regarding the increasing number of identified secondary BAs and the potential effects of BAs-mediated signaling pathways on multiple tissues, it is imperative to have more in-depth studies in the research of the gut microbiota and BAs. Bibliometrics provides quantitative analyses of literature publications via visualization software. This analysis depicts the development trend of the research field, exceptional authors, high-yield research institutions, essential publications, and credible research hotspots (Agarwal et al., 2016). However, to our knowledge, no bibliometric study was conducted to summarize the development trends and future directions of BAs and gut microbiota research. Based on the literature from the Web of Science Core Collection (WoSCC) between 2003 and 2022, our study aimed to provide the publication trends in the studies of gut microbiota and BAs and present the potential hotspots for further research.

## 2 Materials and methods

### 2.1 Data collection

**Figure 1:**
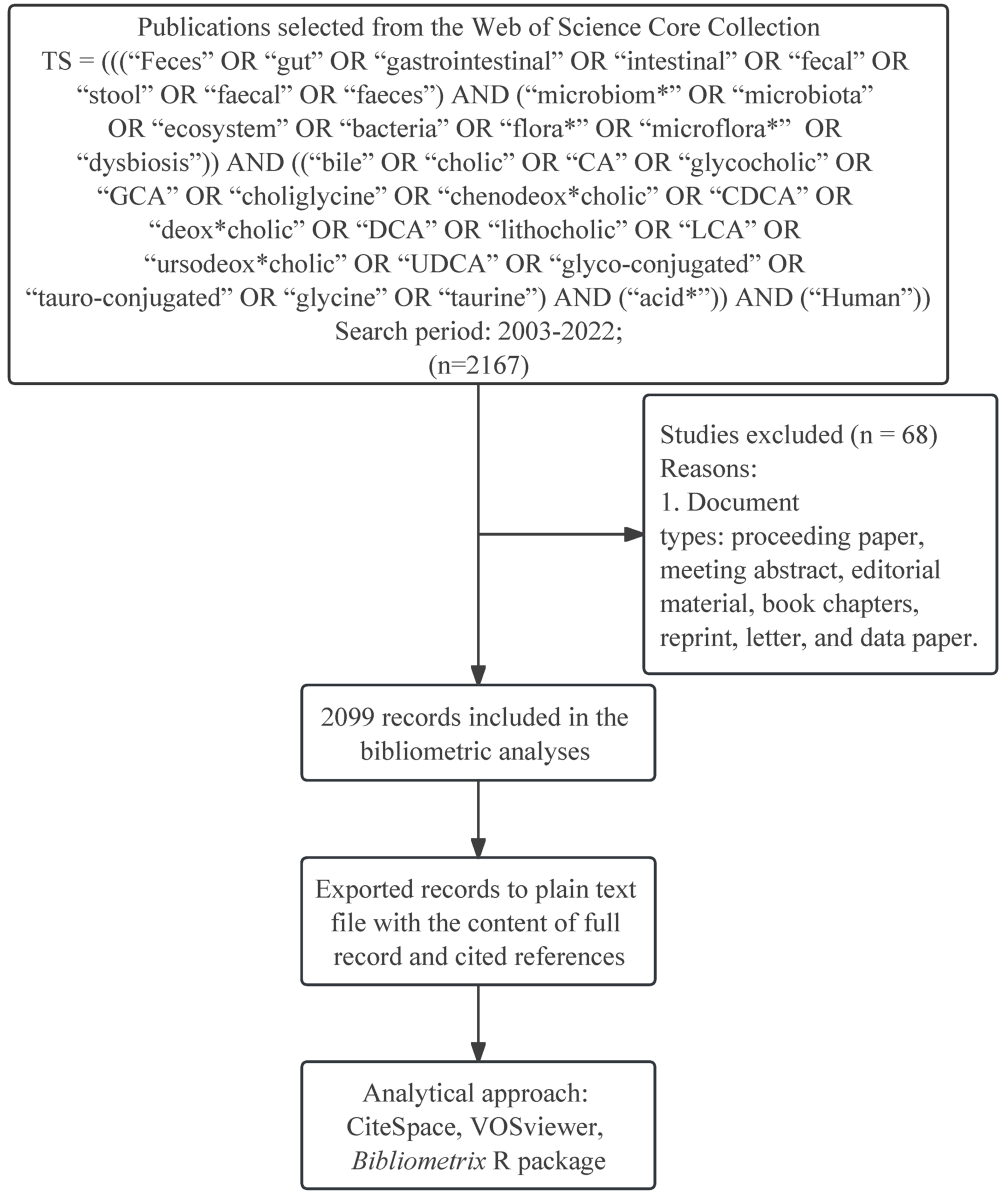
Flow diagram illustrates the search process in gut microbiota and bile acid.

WoSCC provides comprehensive information on article records from high-quality journals worldwide and is considered the most reliable source database for bibliometric analysis (Pei et al., 2022; Ling et al., 2023; Sabe et al., 2023). Thus, we chose WoSCC as the only platform for conducting the following search strategy from 2003 to 2022: TS = (((“Feces” OR “gut” OR “gastrointestinal” OR “intestinal” OR “fecal” OR “stool” OR “faecal” OR “faeces”) AND (“microbiom*” OR “microbiota” OR “ecosystem” OR “bacteria” OR “flora*” OR “microflora*” OR “dysbiosis”)) AND ((“bile” OR “cholic” OR “CA” OR “glycocholic” OR “GCA” OR “choliglycine” OR “chenodeox*cholic” OR “CDCA” OR “deox*cholic” OR “DCA” OR “lithocholic” OR “LCA” OR “ursodeox*cholic” OR “UDCA” OR “glyco-conjugated” OR “tauro-conjugated” OR “glycine” OR “taurine”) AND (“acid*”)) AND (“Human”)). Only reviews and articles were filtered without restricting language to maximize the representation of the retrieved publications. The detailed search strategy can be found in the appendix (Supplementary Table S1). In addition, the flow diagram depicts the procedure for identifying eligible publications (Figure 1). These selected records were exported to plain text files with the content of complete records and cited references.

### 2.2 Bibliometric analysis

We used three bibliometric software packages to visualize the characteristics of enrolled papers in the field of gut microbiota and BAs research, including CiteSpace (version 6.1.R6), VOSview (version 1.6.19), and *Bibliometrix* R package (version 4.1.3). Two techniques are widely used to perform the bibliometric analysis: performance analysis via depicting the metrics in publications and citations and science mapping by constructing the interaction network in the citation, co-citation, bibliographic coupling, co-word, and co-authorship (Donthu et al., 2021). The following metric data of enrolled papers were collected in our study: author name, author affiliations, citations, title, keywords, DOI, references, and journals.

CiteSpace could make graph-based maps with lines between items indicating the relations according to the classification of visualized maps. In contrast, VOSviewer and *Bibliometrix* produce distance-based maps that consider the length of lines as the degree of connectivity of items (van Eck and Waltman, 2010). CiteSpace mainly focuses on identifying the evolution of the knowledge field related to the scientific literature (Chen, 2004; Chen, 2006). We applied this tool to perform burst detection in references and keywords to detect emerging trends and research hotspots. Meanwhile, we illustrated the network of co-cited references, and the relationship between citing journals and cited ones. In addition, VOSviewer is another software applied to construct and visualize bibliometric networks based on the relationship between the items (van Eck and Waltman, 2010). Our study used it to build citations of journals, co-cited references, co-occurrence in keywords, and co-authorship in countries, organizations, and authors. Finally, *Bibliometrix* R, a web-based platform, was utilized to investigate the publications data of country/region (Aria, 2017).

To examine the scientometric information explicitly, we included category normalized citation impact (CNCI), the impact factor (IF), and Hirsch’s index (H-index) in the table, with all indicators derived from the Web of Science.

## 3 Results

### 3.1 Dynamic trends in globally publication outputs and citations

**Figure 2:**
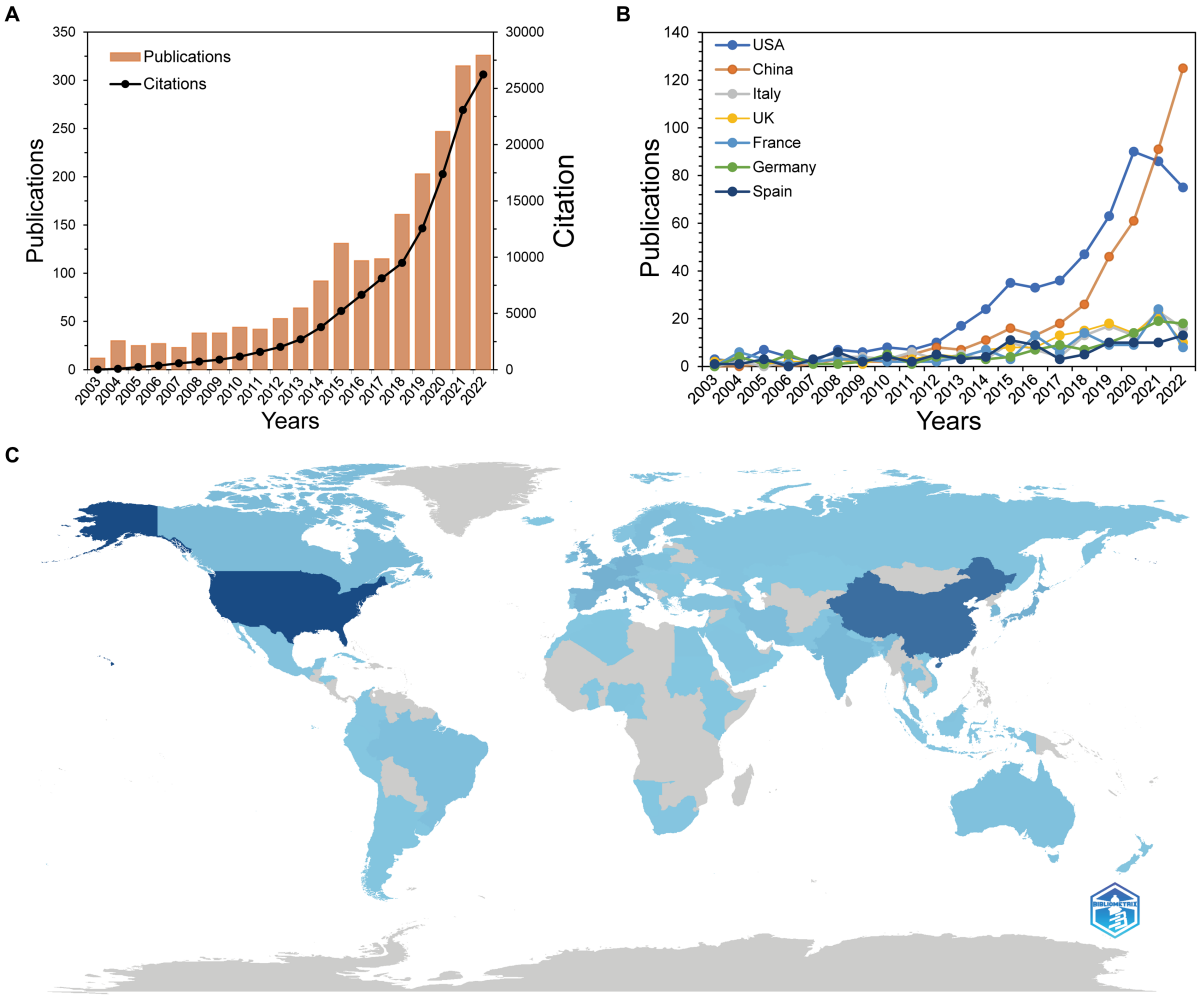
Publication trends of the research on bile acids and gut microbiota. **(A)** Trends of global publications and citations on bile acids research in gut microbiota. **(B)** Trends in top seven productive countries’ publications. **(C)** Geographical distribution map based on the total publications of different countries or regions. The darker the blue on the geographical map is, the more publications the country contributes.

We identified 2099 papers published in gut microbiota and BAs research from WoSCC. Figure 2A depicts the global publication trend and total citations for researching gut microbiota and BAs throughout 2003–2022. This field has evolved dramatically, with an average annual growth rate of 23.31%, especially more than half of enrolled studies published in the last 5 years. Besides, citations of publications have a similar upward trend but with a sharper increase. The total cited times of all articles was 122,790 on 25 September 2023, with an average citation of 58.50 per paper. To pinpoint the countries that contributed the most to the research output, Figure 2B reveals the top seven countries that had >100 papers associated with gut microbiota and BAs. These countries have gradually increased in publication over the past two decades. The USA and China played a pivotal role in the significant increase in studies after 2018. Despite most countries having a slight decline in publications between 2021 and 2022, China surpassed the USA and continued to make a soaring contribution in the targeted research field. Eighty-seven countries or regions have published relevant documents on this topic. Figure 2C illustrates the global geographical distribution of all publications, especially Asia, North America, and European countries making significant contributions.

### 3.2 Analysis of the collaboration between countries or regions

**Figure 3:**
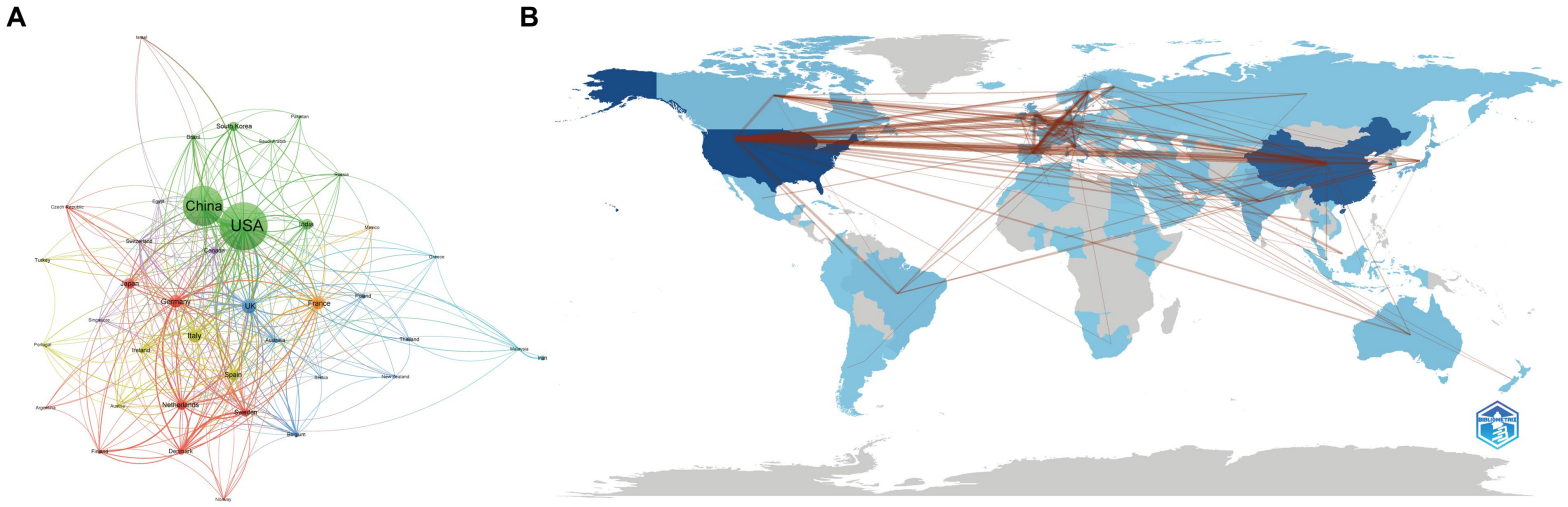
Global cooperation between countries or regions. **(A)** The country’s or regions’ cooperation visualization network generated by VOSviewer. **(B)** Geographical distribution map of the international collaboration generated by the *Bibilometrix* R package. Each node represents the country or region, the node’s size represents the number of publications, and the width of the line between nodes represents the strength of the cooperation. The darker the blue on the geographical map is, the more publications the country contributes.

We used VOSviewer to show the global cooperation of 40 nations when the minimum number of documents published by countries or regions was set as 10 (Figure 3A). According to the total link strength (TLS) representing the level of global co-authorship, the countries with the top five TLS were USA (TLS = 317), UK (TLS = 180), China (TLS = 157), Germany (TLS = 150), and the Netherlands (TLS = 133). Table 1 reveals the top 10 nations/regions for the field of gut microbiota and BAs, which proves that China has the highest 5-year published growth rate, followed by India and the USA. Approximately half of the research outputs were made by the USA (26.77%, 562) and China (20.77%, 436). The CNCI could provide a harmonized h-index across multiple disciplines, which is critical to evaluating the importance of contributions in interdisciplinary research. Supplementary Table S2 reveals that the related research in gut microbiota and BAs involved 56 subjects. According to the H-index and CNCI, the USA has unparalleled dominance in researching gut microbiota and BAs. Although China published the second most papers, receiving the second most citations, the influence and importance of the publications were lower than those of the other seven European countries. Besides, the *Bibliometrix* platform was utilized to evaluate the level of the countries’ collaboration corresponding to the thickness of lines. Moreover, a world map of countries’ collaborations revealed the detailed co-authorship between countries or regions (Figure 3B).

**Table 1:** Top 10 productive countries contributing the most to gut microbiota and bile acid research.

Year	Publications	Percentage	H-index	Total citations	Average citation per paper	CNCI	Number of articles				Growth rate from first to fourth 5-year period (%)
							2003–2007	2008–2012	2013–2017	2018–2022	
World	2099	100%	159	140,164	66.78		117	215	515	1,252	970.09
USA	562	26.77%	115	57,817	102.88	3.92	18	38	145	361	1905.56
China	436	20.77%	58	15,389	35.30	2.03	2	20	65	349	17350.00
UK	146	6.96%	56	15,287	104.71	3.84	11	16	40	79	618.18
Italy	136	6.48%	50	8,122	59.72	2.27	5	20	29	82	1540.00
France	121	5.76%	50	13,899	114.87	2.91	12	12	33	64	433.33
Germany	119	5.67%	38	7,326	61.56	2.64	11	13	27	68	518.18
Spain	105	5.00%	45	9,046	86.15	3.09	8	19	30	48	500.00
India	100	4.76%	29	3,286	32.86	1.22	3	10	26	61	1933.33
Japan	98	4.67%	34	5,604	57.18	2.23	9	9	29	51	466.67
Netherlands	94	4.48%	42	8,484	90.26	3.50	6	5	24	59	883.33

H-index, Hirsch’s index; CNCI, category normalized citation impact.

### 3.3 Analysis of the collaboration between institutions

**Figure 4:**
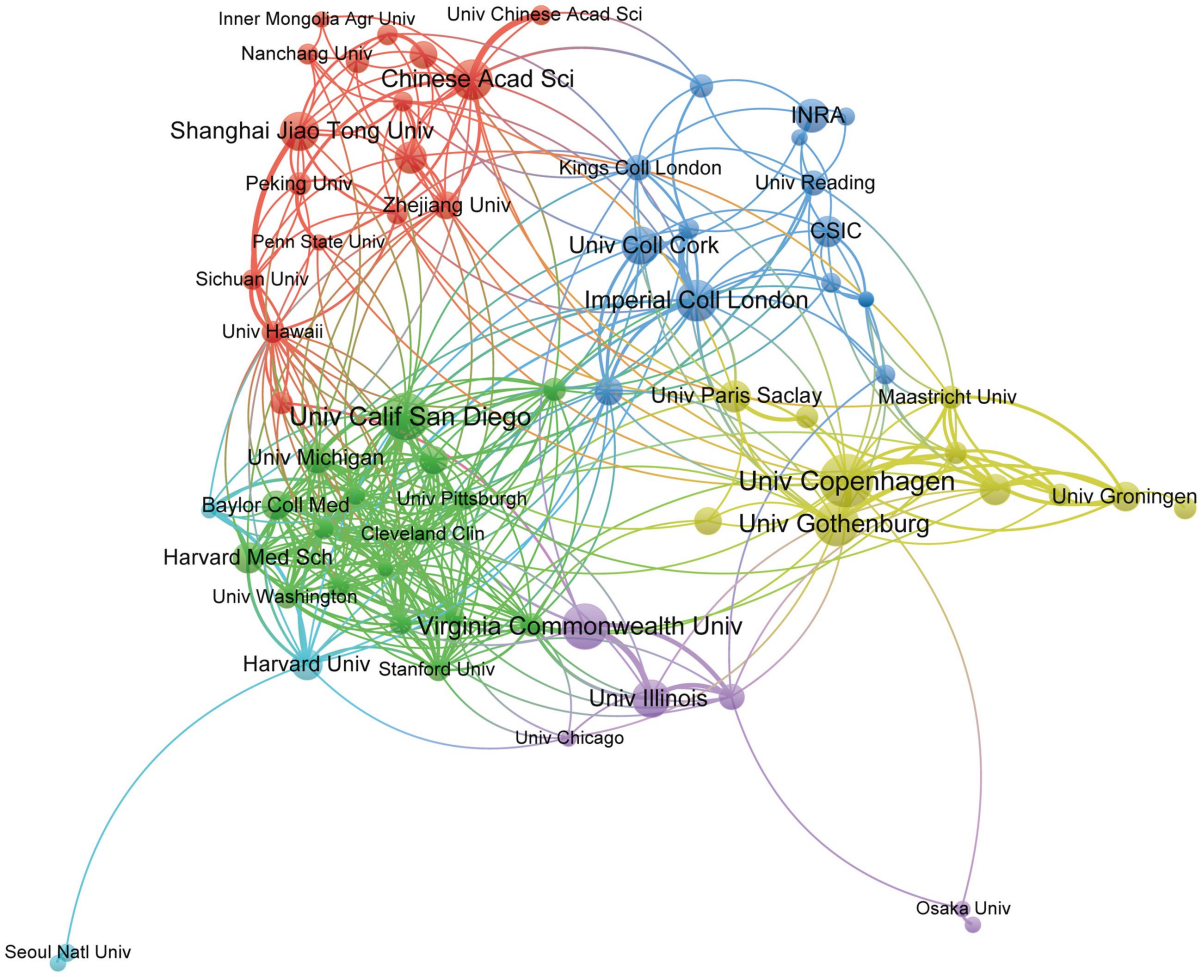
Institutions’ cooperation visualization network generated by VOSviewer. Each node represents the institution, the node’s size represents the number of publications, the width of the line between nodes represents the cooperation’s strength, and the nodes’ color represents different clusters.

Globally, 2,637 institutions have made publications in gut microbiota and BAs research. Table 2 lists the top 10 contributing institutions. Despite the USA and China having the most significant number of institutions engaged in the topic, the University of Copenhagen not only made an enormous contribution but also has played a comparable influence with Imperial College London and Institut National de la Recherche Agronomique (INRA), according to the H-index. The top 10 institutions contributed 307 publications, accounting for 14.63% of papers. Besides, the University of Gothenburg obtained the highest citation times with 33 documents, whereas INRA received the highest average citation per paper. To further pinpoint the specified cooperation among institutions, VOSviewer was applied to visualize the interaction network of 69 institutions, as we set the lowest number of papers by the institution as 10 (Figure 4). The four institutions with the highest TLS were the University of Copenhagen (TLS = 44), Imperial College London (TLS = 42), Brigham & Women’s Hospital (TLS = 41), and Harvard Medical School (TLS = 41). It is worth noting that the University of Copenhagen has a broad collaboration with international institutions in Asia, Europe, and North America.

**Table 2:** Top 10 institutions contribute the most to the gut microbiota and bile acid research.

Institutions	Counts	Country	H-index	TLS	Citation	Average citation per paper
University of Copenhagen	41	Denmark	31	44	9,582	233.71
University of California San Diego	35	USA	24	38	4,921	140.60
Virginia Commonwealth University	34	USA	24	24	4,623	135.97
University of Gothenburg	33	Sweden	29	33	10,212	309.45
Imperial College London	30	UK	32	42	2,657	88.57
Chinese Academy of Sciences	29	China	17	35	2060	71.03
Shanghai Jiao Tong University	28	China	18	22	2,637	94.18
University College Cork	27	Ireland	26	13	1991	73.74
University of Illinois	27	USA	19	22	2,428	89.93
INRA	23	French	30	8	8,645	375.87

H-index, Hirsch’s index; TLS, total link strength; INRA, Institut National de la Recherche Agronomique.

### 3.4 Author and co-cited author analysis

**Figure 5:**
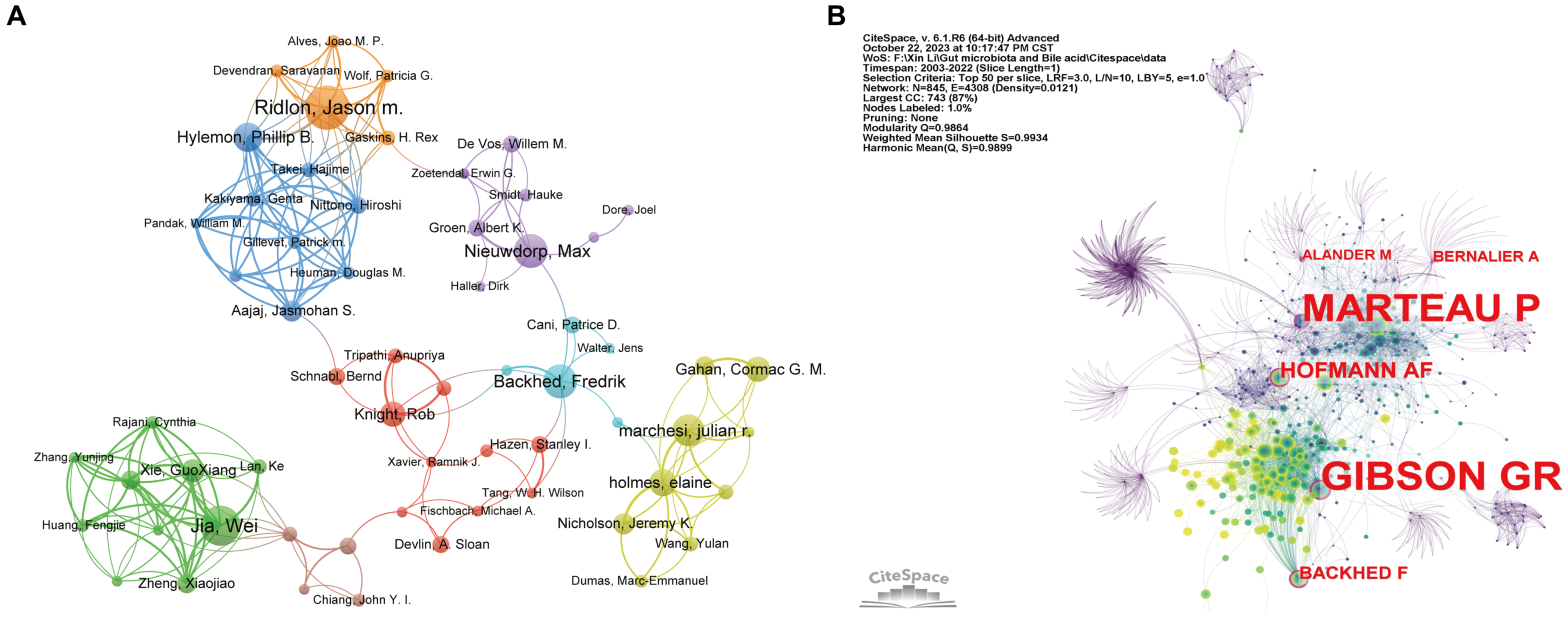
Visualization network of co-authorship and co-citation analyses of authors. **(A)** The co-authorship visualization network of authors generated by VOSviewer. Each node represents the author, the node’s size represents the number of publications, the width of the line between nodes represents the cooperation’s strength, and the nodes’ color represents different clusters. **(B)** The co-citation visualization network of authors generated by CiteSpace. Nodes are shown as an annual ring corresponding to the number of papers the authors published in a given year. The purple circle indicates the authors with the highest centrality value.

A total of 12,154 authors have been involved with the publications of gut microbiota and BAs over the past two decades. To identify the cooperation among authors, we utilized the VOSviewer software to construct the co-occurrence network of 61 authors after setting the minimum number of publications of the author as five (Figure 5A). Five authors with the highest TLS were Wei Jia (TLS = 60), Guoxiang Xie (TLS = 51), Hiroshi Nittono (TLS = 51), Aihua Zhai (TLS = 49), and Phillip b. Hylemon (TLS = 48). The top 10 contributing authors are listed in Table 3, in which Jason M. Ridlon (21 papers), Wei Jia (19 papers), Fredrik Backhed (16 papers), and Max Nieuwdorp (16 papers) made the most publications. Furthermore, we applied CiteSpace to perform the co-citation analysis of authors with the default setting. As a graph-theoretical property, the centrality of each node measures the significance of the node’s position in a network, which provides a tool for identifying the pivotal points between clusters (Chen, 2006). Figure 5B reveals that Fredrik Backhed (0.12) was the only author with centrality >0.10 among the top 10 contributors. Meanwhile, this author had the highest citation times (7,917 times). Therefore, it was proven that Fredrik Backhed has had a considerable impact on the research field of gut microbiota and BAs.

**Table 3:** Top 10 authors contributing the most to the gut microbiota and bile acid research.

Authors	Counts	TLS	Citation	Average citation per paper	H-index	Centrality
Ridlon, Jason M.	21	38	1,625	77.38	19	0.07
Jia, Wei	19	60	2,217	116.68	14	0.00
Backhed, Fredrik	16	13	7,917	494.81	17	0.12
Nieuwdorp, Max	16	19	2,610	163.12	18	–
Marchesi, Julian R.	15	24	1,472	98.13	14	0.00
Hylemon, Phillip b.	14	48	1933	138.07	16	0.01
Holmes, Elaine	13	32	1932	148.62	13	0.00
Gahan, Cormac G. M.	12	12	1,455	121.25	11	0.00
Knight, Rob	12	19	3,663	305.25	10	–
Xie, Guoxiang	11	51	1,319	119.91	11	0.00

TLS, Total link strength; H-index, Hirsch’s index.

### 3.5 Distribution of subjects and source journals

Supplementary Table S2 shows that the top five subjects were Microbiology, Food Science and Technology, Biochemistry and Molecular Biology, Biotechnology and Applied Microbiology, Gastroenterology, and Hepatology. Additional disciplines involved in our enrolled publications included nutrition and dietetics (184, 8.77%), pharmacology and pharmacy (120, 5.72%), multidisciplinary sciences (120, 5.72%), endocrinology and metabolism (102, 4.86%), and other subjects.

**Figure 6:**
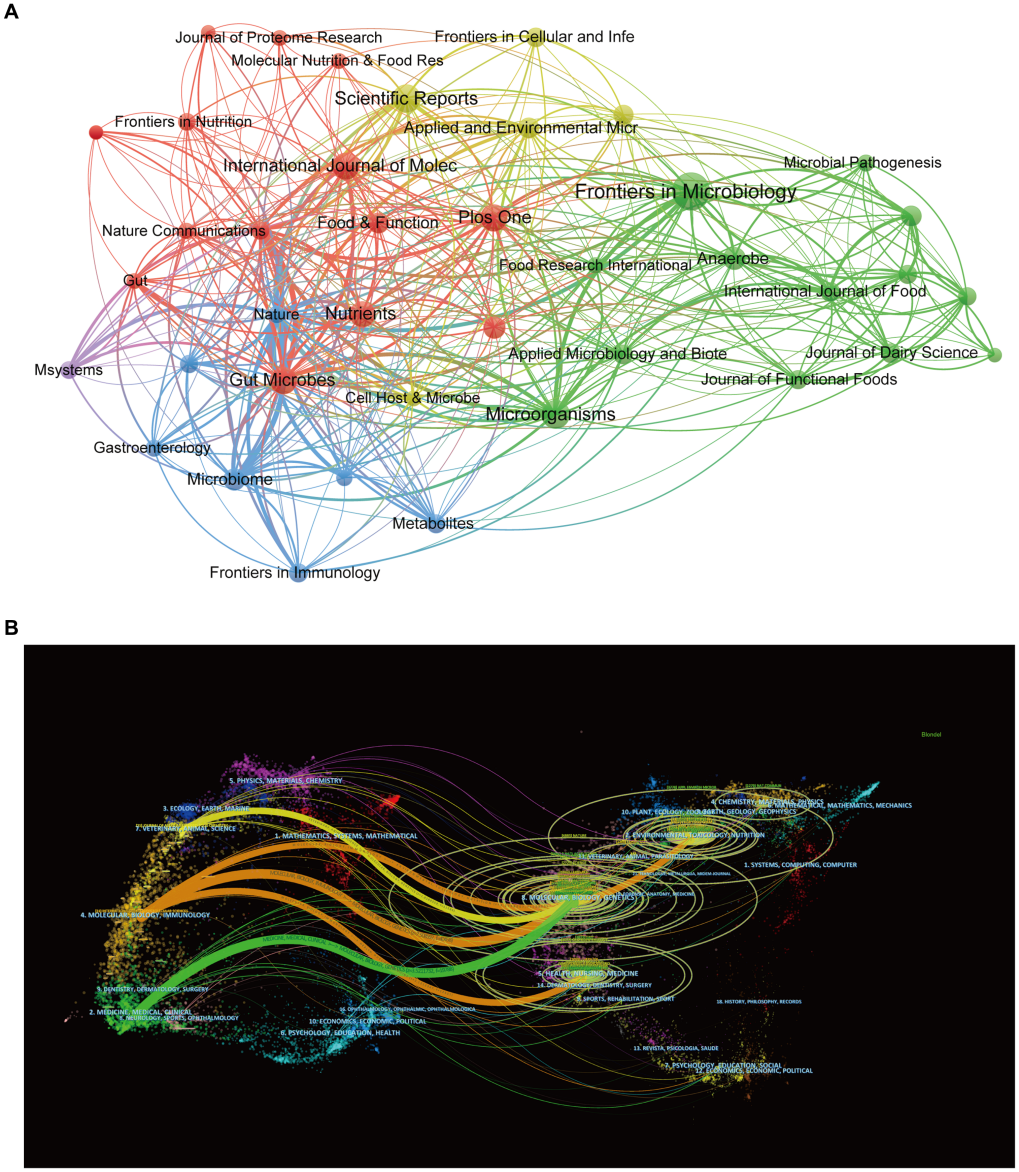
Visualization network of source journals. **(A)** Co-occurrence visualization network of journals generated by VOSviewer. Each node represents the source journal, the node’s size represents the number of publications, the width of the line between nodes represents the citation’s strength, and the nodes’ color represents different clusters. **(B)** A dual-map overlay of journals associated with bile acids in gut microbiota. Each node represented a journal that was classified into different disciplines and marked by various colors. The size of nodes corresponded to the volume of publications of journals. The width of the line between nodes represents the frequency of z-score-scale citation.

According to Figure 6A, Frontiers in Microbiology (*n* = 59), Scientific Reports (*n* = 35), and Microorganisms (*n* = 34) are the top three academic publications that publish studies on gut microbiota and BAs out of 742 journals, making up 6.10% of all publications. To further reflect the influence of the research on gut microbiota and BAs, journal citations were considered the most essential metrics. Table 4 lists the top 10 journals with the highest citation times. Nature had the highest IF, followed by Cell Host & Microbe (IF 2022 = 30.3) and Gastroenterology (IF 2022 = 29.4). Seven of the 10 journals were categorized as Q1 (the top 25% of the IF distribution) in the quartile category. Notably, the highest citation times (15,309), the highest average amount of citations per publication (1093.50), and the highest IF (IF 2022 = 64.8) are all attributes of Nature. Therefore, these findings suggest that journals such as Nature have a critical role in promoting the advancement of the topic.

**Table 4:** Top 10 highest cited journals.

Journals	Counts	IF (2022)	JCR (2022)	Citations	Average citation per paper
Nature	14	64.8	Q1	15,309	1093.50
Journal of Lipid Research	15	6.5	Q1	2,931	195.40
PLoS One	31	3.7	Q2	2,754	88.84
Cell Host & Microbe	11	30.3	Q1	2,245	204.09
International Journal of Food Microbiology	17	5.4	Q1	2,063	121.35
Frontiers In Microbiology	59	5.2	Q2	1,973	33.44
British Journal of Nutrition	18	3.6	Q3	1,932	107.33
Gastroenterology	13	29.4	Q1	1,920	147.69
Gut Microbes	31	12.2	Q1	1,917	61.84
Gut	11	24.5	Q1	1,793	163.00

IF, impact factor; JCR, Journal Citation Reports.

Additionally, Figure 6B depicts the distribution landscape of the journal’s topics in the dual-map overlay of journals. The enrolled journals covered the various study areas labeled on the map. The citing and cited journals appeared on the left and right sides of the map, respectively. According to the width of the reference paths, Molecular/Biology/Immunology and Medicine/Medical/Clinical journals are significantly interconnected with Molecular/Biology/Genetics journals, indicating high degree of interdisciplinary integration between these disciplines in the research of gut microbiota and bile acid.

### 3.6 Analysis of co-cited references

**Figure 7:**
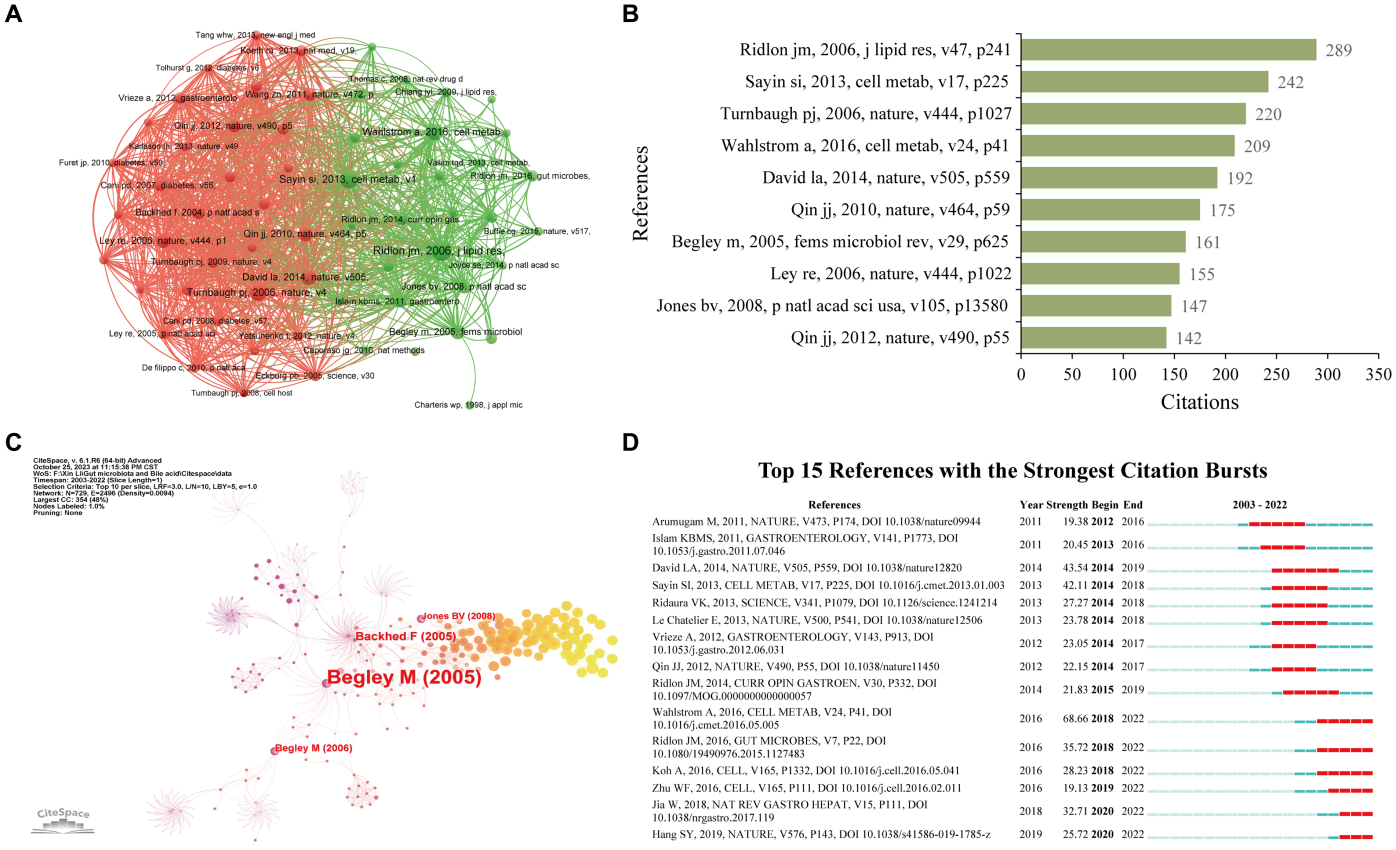
Co-citation network of cited references. **(A)** Co-citation visualization network of cited references generated by VOSviewer. Each node represents the cited references, the node’s size represents the citation times, the width of the line between nodes represents the co-citation times, and the color of the nodes represents different clusters. **(B)** Histogram illustrating the citation times of the top 10 cited references. **(C)** Co-citation network of cited references generated by CiteSpace. Nodes are shown as an annual ring corresponding to the citation times of each cited reference received in a given year. The purple circle indicates the cited reference with a higher centrality value. **(D)** Citation burst analysis of cited references generated by CiteSpace.

Enrolled publications have cited 91,193 references in total. We used VOSviewer to perform the co-citation analysis of cited references with a minimum citation number of 65. Figure 7A reveals the co-citation network of 62 cited references. It is proven that Turnbaugh PJ, 2006, Nature (Turnbaugh et al., 2006) has the highest amount of co-citation with other references (2164), followed by Sayin Si, 2013, Cell Metabolism (Sayin et al., 2013) (2012), and Ridlon JM, 2006, Journal of Lipid Research (Ridlon et al., 2006) (1856). Meanwhile, Table 5 lists the top 10 most cited references in the research on gut microbiota and BAs. Ridlon JM, 2006, Journal of Lipid Research (Ridlon et al., 2006) (289), Sayin Si, 2013, Cell Metabolism (Sayin et al., 2013) (242), and Turnbaugh PJ, 2006, Nature (Turnbaugh et al., 2006) (220) have the top number of citations (Figure 7B). Therefore, these studies have a vast impact on this field. Meanwhile, to identify the references with a centrality >0.10, CiteSpace was conducted to construct the co-citation network of references using the top 10% of most cited references (Figure 7C). From the top 10 references, only Begley M, 2005, fems microbiol rev (Begley et al., 2005), and Jones BV (2008), P Natl Acad Sci USA (Jones et al., 2008) were regarded as the critical publications for the advancement of this research. Besides, Figure 7D reveals the top 15 references with the strongest citation bursts. The citations of this research started to increase significantly in 2012. Six references were highly cited in the past 5 years, indicating that gut microbiota and BAs remained a popular research area.

**Table 5:** Top 10 most cited references in gut microbiota and bile acid.

Title	First author	JCR (2022)	IF (2022)	Type	Year	Journal	TLS	Citations
Bile salt biotransformations by human intestinal bacteria	Ridlon, JM	Q1	6.5	Review	2006	Journal of Lipid Research	1856	289
Gut microbiota regulates bile acid metabolism by reducing the levels of tauro-beta-muricholic acid, a naturally occurring FXR antagonist	Sayin, SI	Q1	29	Article	2013	Cell Metabolism	2012	242
An obesity-associated gut microbiome with increased capacity for energy harvest	Turnbaugh, PJ	Q1	64.8	Article	2006	Nature	2,164	220
Intestinal cross-talk between Bile Acids and Microbiota and Its Impact on Host Metabolism	Wahlstrom, A	Q1	29	Review	2016	Cell Metabolism	1,290	209
Diet rapidly and reproducibly alters the human gut microbiome	Aavid, LA	Q1	64.8	Article	2014	Nature	1,630	192
A human gut microbial gene catalog established by metagenomic sequencing	Qin, JJ	Q1	64.8	Article	2010	Nature	1,434	175
The interaction between bacteria and bile	Begley, M	Q1	11.3	Review	2005	Fems Microbiology Reviews	882	161
Microbial ecology: human gut microbes associated with obesity	Ley, RE	Q1	64.8	Article	2006	Nature	1,678	155
Functional and comparative metagenomic analysis of bile salt hydrolase activity in the human gut microbiome	Jones, BV	Q1	11.1	Article	2008	PNAS	1,054	147
A metagenome-wide association study of gut microbiota in type 2 diabetes	Qin, JJ	Q1	64.8	Article	2012	Nature	1,459	142

JCR, Journal citation reports; IF, impact factor; TLS, total link strength.

### 3.7 Analysis of keywords for the research hotspots

**Figure 8:**
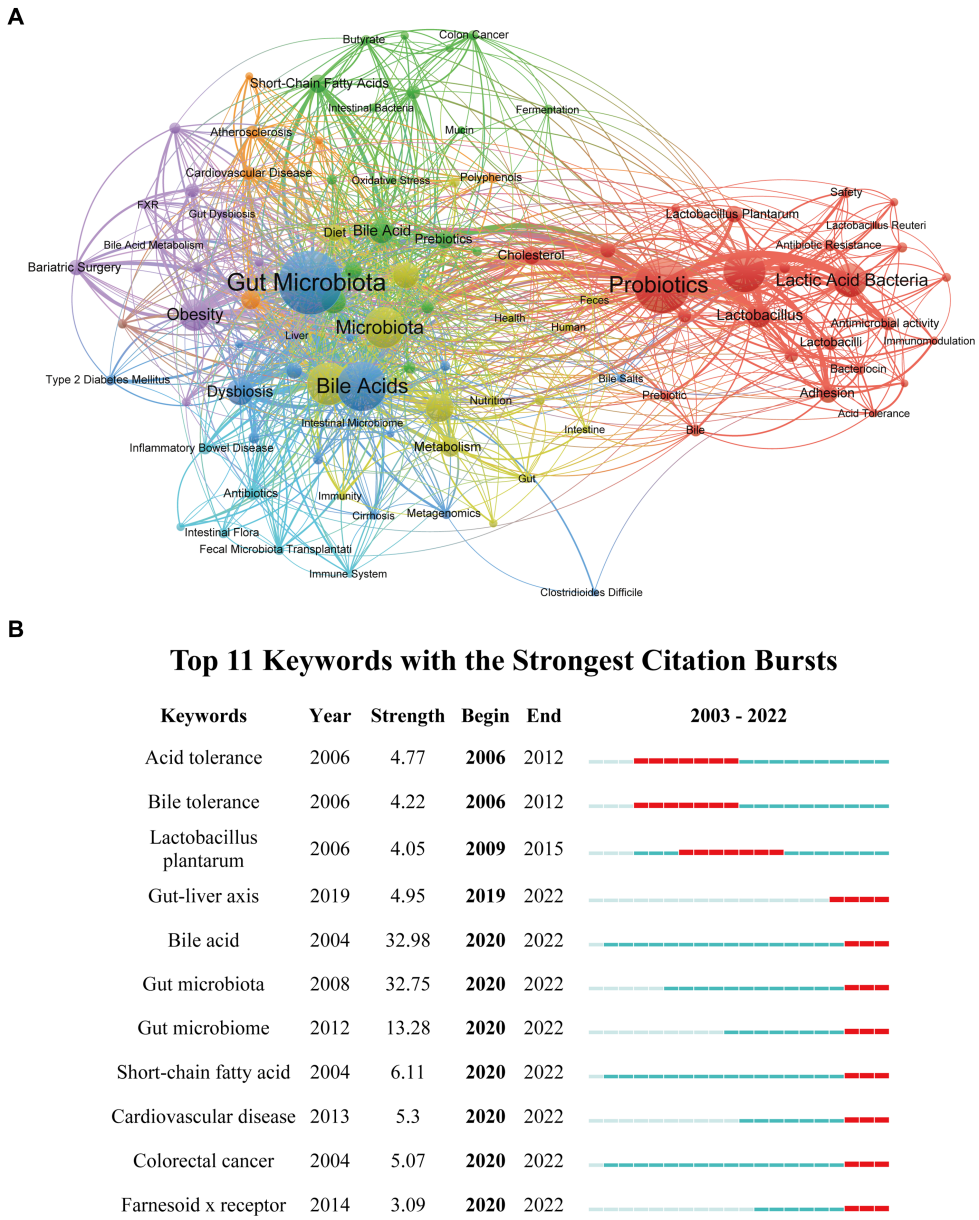
Visualization network of keywords co-occurrence and keyword citation burst analysis. **(A)** The visualization network of keywords co-occurrence generated by VOSviewer. Each node represents the keyword, the node’s size represents the number of occurrences, the width of the line between nodes represents the number of co-occurrences, and the color of the nodes represents different clusters. **(B)** Keywords burst analysis generated by CiteSpace.

A total of 3,989 author keywords in our study were collectively analyzed to identify the intensified research areas and potential research frontiers in gut microbiota and BAs. The co-occurrence network constructed by VOSviewer included 100 keywords, with the minimum number of keyword occurrences set to 10 (Figure 8A). It was displayed that these keywords were categorized into eight clusters (green, orange, purple, dark blue, light blue, red, yellow, and brown). Furthermore, Table 6 lists the top 20 keywords with the highest occurrences, which could contribute to pinpointing the established research hotspots and newly developed areas. Thus, probiotics, microbiome, lactic acid bacteria, and obesity were some keywords with high frequency of occurrences. In addition, CiteSpace was applied to perform the keyword burst analysis for finding potential hotspots and new research directions (Chen, 2006; Pei et al., 2022). In total, 11 top keywords were identified with the earliest starting time in 2006, which also showed the dynamic shift of research direction in this field (Figure 8B). It is worthwhile noting that seven of them have the highest citation time between 2020 and 2022, namely “gut-liver axis” (2019–2022), “bile acid” (2020–2022), “gut microbiota” (2020–2022), “gut microbiome” (2020–2022), “short-chain fatty acid” (2020–2022), “cardiovascular disease” (2020–2022), “colorectal cancer” (2020–2022), and “farnesoid x receptor” (2020–2022), indicating that these terms are widely investigated and recognized at present time.

**Table 6:** Top 20 keywords with the highest occurrence in the field of gut microbiota and bile acid.

Rank	Keywords	TLS	Occurrence	Rank	Keywords	TLS	Occurrence
1	gut microbiota	332	276	11	gut microbiome	84	69
2	probiotics	272	223	12	lactobacillus	81	65
3	bile acids	257	187	13	inflammation	126	63
4	microbiome	229	147	14	bile acid	76	63
5	probiotic	135	147	15	metabolites	67	43
6	microbiota	192	139	16	metabolism	65	43
7	lactic acid bacteria	108	103	17	adhesion	49	42
8	obesity	180	92	18	short-chain fatty acids	84	41
9	metabolomics	82	76	19	cholesterol	62	39
10	dysbiosis	137	71	20	prebiotics	75	37

TLS, Total link strength.

## 4 Discussion

### 4.1 General information

To comprehensively illustrate the global scientific research in gut microbiota and BAs, we performed bibliometric analysis on the enrolled 2099 documents published between 2003 and 2022. Quantitative analysis was applied to the annual publications, affiliated countries, institutions, authors, subjects, and journals. Even though the publication outputs in this field only had an incremental rise from 2003 to 2013, an enormous increase in contributions was seen after 2014, especially more than half of enrolled papers published in the last 5 years, mainly attributed to the study of the USA and China. Meanwhile, the upward trend of citations for the included publications is much more evident. Thus, this area has attracted broad attention from the scientific community, hinting that the research on gut microbiota and BAs is promising. Compared to other countries or regions, the USA and China have made the most publications and received the correspondingly highest total citations in this field. According to the CNCI, H-index, and co-authorship networks among countries, the USA not only plays a dominant role in advancing research in gut microbiota and BAs but also has widespread collaboration with international communities. In addition, five of the top 10 most productive institutions were from the USA and China, and the University of Copenhagen has made the most publications and collaborations with global organizations. INRA and the University of Gothenburg have the highest average citations in each publication.

Among the top 10 contributing authors, Jason M. Ridlon (*n* = 21) has had the most publications, followed by Wei Jia (*n* = 19), Fredrik Backhed (*n* = 16), and Max Nieuwdorp (*n* = 16), which proved their excellent contribution in the field of gut microbiota and BAs. However, based on the centrality value of the co-cited author network, Fredrik Backhed has the most considerable impact on the development of this area. Prof. Fredrik Backhed from the University of Gothenburg and the University of Copenhagen is the author with the highest average citation per paper. Prof. Backhed F. reviewed the role of BAs and gut microbiota in host metabolism and metabolic diseases (Backhed, 2011; Khan et al., 2014; Wu et al., 2015; Arora and Backhed, 2016; Tang et al., 2019). Meanwhile, *in vivo* studies proved that weight loss by bariatric surgery was mainly achieved via regulating gut microbiota and BA–FXR pathways (Ryan et al., 2014; Tremaroli et al., 2015; Wahlström et al., 2017). The clinical study suggested fecal microbiota transplantation could transfer poor donor metabolic traits (de Groot et al., 2020). In addition, based on the distribution of subjects in Supplementary Table S2, the top five subjects with the most articles about gut microbiota and BAs were Microbiology (515, 24.54%), Food Science and Technology (281, 13.39%), Biochemistry and Molecular Biology (233, 11.10%), Biotechnology and Applied Microbiology (218, 10.39%), and Gastroenterology and Hepatology (205, 9.77%). As the journals publishing the most papers in the field, Frontiers in Microbiology (*n* = 59), Scientific Reports (*n* = 35), and Microorganisms (*n* = 34) were probably the primary journals for articles on gut microbiota and BAs. To reflect the influence of this field on the scientific community, the top 10 journals with the highest citation times are displayed in Table 4. Half of these journals had an IF higher than 10.0, including Nature (*n* = 14), Cell Host & Microbe (*n* = 11), Gastroenterology (*n* = 13), Gut (*n* = 11), and Gut Microbes (*n* = 31). Meanwhile, among the other five journals, the Journal of Lipid Research (*n* = 15), International Journal of Food Microbiology (*n* = 17), and Frontiers in Microbiology have an IF between 5.0 and 10.0. It indicated that many high-quality journals published studies in this field.

According to the centrality value >0.10 of the co-citation network of references, two publications were considered the most significant studies on the development of research between gut microbiota and BAs, namely Begley M, 2005, fems microbiol rev (Begley et al., 2005), and Jones BV, 2008, P Natl Acad Sci USA (Jones et al., 2008). Begley M et al. systematically reviewed the relevant studies about the cross-talk between different types of bacteria and BAs from the perspective of antimicrobial actions of bile, genetics of bacterial bile tolerance, and bile-related pathogenesis of bacteria (Begley et al., 2005). It also pointed to the imperative need for research on the molecular mechanisms of how gut bacteria regulate bile tolerance and the relationship between bile and intestinal pathogenesis. Furthermore, Jones BV et al. first reported that bile salt hydrolases (BSHs) were enriched in the human gut microbiome, which could mediate bile tolerance *in vitro*, promoting bacterial survival in the murine gut *in vivo* (Jones et al., 2008). According to the top 15 references with the highest burst signal, 6 references spanning the time until 2022 are primarily associated with the interaction of gut microbiota and BAs on immune responses, gastrointestinal carcinogenesis, and metabolism in the host.

### 4.2 Hotspots and research frontiers in gut microbiota and bile acid

Keywords with high frequency can indicate the hot research direction and emerging frontiers in gut microbiota and BAs. We applied the keywords citation burst analysis of CiteSpace to foretell the future direction of this research. Thus, five research areas with strong citation burst until 2022 were principally identified in our study: “gut-liver axis,” “short-chain fatty acid,” “cardiovascular disease,” “colorectal cancer,” and “Farnesoid x receptor.”

#### 4.2.1 Gut–liver axis

The gut–liver axis emerging as a focus research field has been considered the bidirectional interaction and cross-talk between the intestine and the liver (Kim et al., 2023). Bioactive mediators synthesized by the liver are secreted into the intestine via the biliary tract, which could impact the composition of the gut microbiome and gut barrier integrity. Meanwhile, the portal vein could translocate intestinal metabolites into the liver, leading to changes in BAs synthesis and glucose and lipid metabolism of the liver (Tripathi et al., 2018). Previous studies proved that the gut–liver axis is closely associated with the pathogenesis of a spectrum of liver diseases, including non-alcoholic fatty liver disease (NAFLD), alcoholic liver disease, primary sclerosing cholangitis (PSC), cirrhosis, and hepatocellular carcinoma (HCC) (Marra and Svegliati-Baroni, 2018; Tripathi et al., 2018; Albillos et al., 2020; Kim et al., 2023; Rajapakse et al., 2023). As the main biomolecules synthesized in the liver, BAs closely cross-talk with the gut microbiota via the gut–liver axis. Previous studies reported that the level of plasma BAs and hepatic BAs was significantly increased in non-alcoholic steatohepatitis patients, which could induce cytotoxicity and be involved with the pathogenesis of NAFLD (Aranha et al., 2008; Ferslew et al., 2015). As one of the cholestatic liver diseases, PSC has decreased bile flow via the biliary tract, contributing to the elevated plasma level of conjugated and unconjugated primary BAs (Kim et al., 2023). Due to compromised hepatocyte function in cirrhosis patients, primary BAs secreted into the gut are significantly reduced (Kakiyama et al., 2013), contributing to the reduced total fecal BAs with a decreased secondary to primary BAs and high serum primary BAs (Kakiyama et al., 2013). Besides, secondary BAs contribute to an immunosuppressive tumor microenvironment by inhibiting NKT cell infiltration and promoting the polarization of M2-like tumor-associated macrophage, which could lead to the progression of HCC (Ma et al., 2018; Sun et al., 2022).

Therefore, most research on BAs in liver diseases is derived from observational studies. The molecular mechanism of the BAs in the pathogenesis of these disorders via the gut–liver axis remains unelucidated, and intensive research is needed in the future.

#### 4.2.2 Short-chain fatty acids

Short-chain fatty acids (SCFAs) are primarily produced from the microbial fermentation of dietary fiber and other fermentable carbohydrates (Krautkramer et al., 2021). Acetate, propionate, and butyrate are the dominant components of SCFAs in the gut of humans (den Besten et al., 2013). Accumulating studies reported that SCFAs are associated with the development of cardiovascular diseases (CVDs), metabolic disorders, and cancer (Nogal et al., 2021; Jaye et al., 2022). Pluznick et al. (2013) reported that SCFAs could regulate blood pressure via receptors Olfr78 and FFAR3. Furthermore, by activating receptors FFAR2 and FFAR3, SCFAs could enhance glucose homeostasis by controlling blood glucose levels and promoting glucose uptake (Nogal et al., 2021). Meanwhile, propionates reduce plasma cholesterol by inhibiting endogenous lipolysis and promoting the hepatic uptake of circulating cholesterol (Al-Lahham et al., 2012; Nguyen et al., 2019). Besides, butyrate could exert a tumor-suppressive effect on colorectal cancer (CRC) via the histone hyperacetylation-mediated pathway and GPR43 receptor signaling pathways (Tang et al., 2011; Luu and Visekruna, 2019).

However, the detailed molecular mechanisms of SCFAs in cardio-metabolic diseases remain unclear. Research about the impact of SCFAs on non-CRC carcinomas is also needed.

#### 4.2.3 Cardiovascular diseases

CVDs are the leading cause of death in Western countries (Mozaffarian et al., 2016). It is widely demonstrated that gut microbiota and its metabolites are correlated with the pathogenesis of CVDs (Anselmi et al., 2021). Conjugated BAs can activate the sphingosine-1-phosphate receptor 2 (S1PR2) signaling to promote the accumulation of atherosclerotic plaque in the apolipoprotein E-deficient mouse model (Skoura et al., 2011). Mayerhofer et al. (2017) reported that low levels of primary and secondary BAs are associated with worse overall survival in patients with chronic heart failure. Meanwhile, lower serum levels of total BAs could indicate the presence and severity of coronary artery disease (Li et al., 2020). In addition, secondary BAs can reduce cholesterol levels by activating FXR and TGR5 (Jia et al., 2023), which contribute to the beneficial effect on the outcome of CVD. Therefore, an unbalanced composition of BAs could potentially increase cholesterol and CVD progression (Kazemian et al., 2020). However, the impact of changes in BA profiles on the development and therapeutic responses of CVDs remains largely unknown.

#### 4.2.4 Colorectal cancer

CRC is one of the most diagnosed malignancies and the leading cause of cancer death globally (Keum and Giovannucci, 2019). Several studies reported that BAs were involved in the carcinogenesis of CRC (Bernstein et al., 2011; Cao et al., 2017). The plasma level of BAs is positively associated with the risk of colon cancer (Kühn et al., 2020). Fu et al. (2019) explicitly demonstrated that a high-fat diet could promote the adenoma-to-adenocarcinoma progression in a mouse model of CRC via promoting the proliferation of intestinal stem cells and decreasing chromosome stability, which attributed to the upregulated plasma level of TβMCA and DCA. Besides, LCA plays a tumor-promoting role in CRC by inhibiting apoptosis, enhancing cell proliferation, reducing oxidative DNA damage, and activating the NK-*κ*B signaling pathway (Sinha et al., 2020). DCA also has a tumor-promoting function in CRC by stimulating the extracellular signal-related kinase (ERK) signaling pathway and inducing membrane-perturbing effects (Qu et al., 2023). In addition, UDCA as secondary BAs could inhibit the progression of CRC via repressing cancer cell proliferation (Kim et al., 2017), downregulating cyclooxygenase 2 (COX2) transcription (Khare et al., 2008), and decreasing DCA-induced inflammation (Shah et al., 2006). However, most *in vivo* studies are based on small intestine tumors derived from Apc^min/+^ mice, which could lead to biased results in CRC. The therapeutic application of BAs on CRC patients is immensely needed in the clinical trial.

#### 4.2.5 Farnesoid x receptor

Whole-body BA homeostasis is primarily regulated by the Farnesoid x receptor (FXR)-targeted bile acid metabolizing enzymes and transporters (Hofmann and Hagey, 2014). Thus, it is demonstrated that FXR participated in the pathogenesis of a wide range of cardio-metabolic diseases and gastrointestinal carcinomas. *In vivo* studies proved that the inhibition of intestinal FXR signaling could reduce obesity, insulin resistance, and fatty liver disease by regulating enterohepatic BA metabolism and intestinal ceramide synthesis (Cariou et al., 2006; Prawitt et al., 2011; Gonzalez et al., 2016). Due to the repressive effect of FXR activation on BA synthesis, the agonist of FXR could decrease the abnormally high levels of hepatic BAs in cholestatic liver diseases (Hirschfield et al., 2015), which contributed to the approval of obeticholic acid in the treatment of PSC by the FDA (Markham and Keam, 2016). Besides, previous results have pointed out that FXR could inhibit the progression of atherosclerotic plaques by promoting cholesterol excretion to the gut lumen and inhibiting cholesterol absorption (Hartman et al., 2009; de Boer et al., 2017; Li et al., 2019). Intestinal levels of FXR are significantly downregulated in CRC patients, and its expression has an inverse correlation with the progression of CRC, mainly attributed to DNA methylation and KRAS signaling (Sun et al., 2021). Furthermore, FXR has a tumor suppressor effect on CRC via interacting with β-catenin and inhibiting the transcription of MMP7 (Peng et al., 2019; Yu et al., 2020). Compared to intestinal FXR, hepatic FXR has a comparable tumor suppressor effect on HCC. Liu et al. proved that FXR could disrupt the β-catenin-TCF4 complex via binding β-catenin, leading to the inhibition of HCC (Liu et al., 2015). In addition, synergistic effects between BA accumulation and FXR deficiency are implicated in the spontaneous HCC in global FXR-null mice (Sun et al., 2021). Therefore, the application of tissue-specific FXR-targeted agents is more suitable to investigate.

In summary, gut–liver axis, SCFAs, CVD, CRC, and FXR are the research frontiers in gut microbiota and BAs.

### 4.3 Limitations

First, our study only included the articles published in high-quality journals of the WoSCC database, which led to the neglect of many studies from other databases. Second, bibliometric analysis failed to assess the quality of enrolled studies, which may introduce confounding factors into our results. Third, due to the citation-relevant metrics having time-dependent features, newly published articles may have fewer citations than earlier papers, causing potentially biased findings. Although these limitations may slightly bias the overall results, the primary trend of the field is not impacted in this paper. Therefore, our study presents a complete scenario for comprehending the development trends, research topics, and hotspots in gut microbiota and BAs.

## 5 Conclusion

This study comprehensively investigated the research field of gut microbiota and BAs with rigorous bibliometrics. Our analyses showed that this field has gained enormous attention across different disciplines, especially in the past 5 years. The identified research frontiers could be the focus of subsequent studies about the molecular mechanisms and potential applications of this research in cardiometabolic disorders and gastrointestinal cancers. Therefore, our bibliometric study provided an integrated overview of the gut microbiota and BAs and pinpointed future research directions.

## Data Availability

The original contributions presented in the study are included in the article/Supplementary material, further inquiries can be directed to the corresponding authors.
