# Supplementary material for: Bibliometric analysis of research on gut microbiota and bile acids: publication trends and research frontiers
Source: Front Microbiol. 2024 Aug 21;15:1433910. doi: 10.3389/fmicb.2024.1433910 (PMC11371755; doi:10.3389/fmicb.2024.1433910)
Supplement: Supplementary file 1 [file Table_1.DOCX]

Table S1 Search strategy of identifying publications in the field of bile acid and gut microbiota in Web of Science.

| Step | Search Strategy | Number of Publications |
| --- | --- | --- |
| #1 | (((((((TS=(Feces)) OR TS=(gut)) OR TS=(gastrointestinal)) OR TS=(intestinal)) OR TS=(fecal)) OR TS=(stool)) OR TS=(faecal)) OR TS=(faeces) | 898,427 |
| #2 | ((((((TS=(microbiom*)) OR TS=(microbiota)) OR TS=(ecosystem)) OR TS=(bacteria)) OR TS=(flora*)) OR TS=(microflora*)) OR TS=(dysbiosis) | 1,409,564 |
| #3 | #2 AND #1 | 176,985 |
| #4 | (((((((((((((((((TS=(bile)) OR TS=(cholic)) OR TS=(CA)) OR TS=(glycocholic)) OR TS=(GCA)) OR TS=(choliglycine)) OR TS=(chenodeox*cholic)) OR TS=(CDCA)) OR TS=(deox*cholic)) OR TS=(DCA)) OR TS=(lithocholic)) OR TS=(LCA)) OR TS=(ursodeox*cholic)) OR TS=(UDCA)) OR TS=(glyco-conjugated)) OR TS=(tauro-conjugated)) OR TS=(glycine)) OR TS=(taurine) | 825,425 |
| #5 | TS=(acid*) | 4,201,102 |
| #6 | #5 AND #4 | 203,666 |
| #7 | #3 AND #6 | 8,364 |
| #8 | TS=(Human) | 4,651,153 |
| #9 | #7 AND #8 | 2,572 |
| #10 | (#9) AND FPY=(2003-2022) | 2,167 |
| #11 | (#9) AND FPY=(2003-2022) and Editorial Material or Proceeding Paper or Meeting Abstract or Reprint or Letter or Book Chapters or Data Paper (Exclude – Document Types) | 2,099 |

Annotation: The search strategy was performed on 17 September 2023.
