# Supplementary material for: Bibliometric analysis of research on gut microbiota and bile acids: publication trends and research frontiers
Source: Front Microbiol. 2024 Aug 21;15:1433910. doi: 10.3389/fmicb.2024.1433910 (PMC11371755; doi:10.3389/fmicb.2024.1433910)
Supplement: Supplementary file 2 [file Table_2.DOCX]

Table S2 Distribution of subjects in the research field of gut microbiota and bile acid.

| **Subjects** | **Count** | **Percentage** |
| --- | --- | --- |
| MICROBIOLOGY | 515 | 24.54% |
| FOOD SCIENCE & TECHNOLOGY | 281 | 13.39% |
| BIOCHEMISTRY & MOLECULAR BIOLOGY | 233 | 11.10% |
| BIOTECHNOLOGY & APPLIED MICROBIOLOGY | 218 | 10.39% |
| GASTROENTEROLOGY & HEPATOLOGY | 205 | 9.77% |
| NUTRITION & DIETETICS | 184 | 8.77% |
| PHARMACOLOGY & PHARMACY | 120 | 5.72% |
| MULTIDISCIPLINARY SCIENCES | 120 | 5.72% |
| ENDOCRINOLOGY & METABOLISM | 102 | 4.86% |
| IMMUNOLOGY | 89 | 4.24% |
| CELL BIOLOGY | 77 | 3.67% |
| MEDICINE, RESEARCH & EXPERIMENTAL | 67 | 3.19% |
| CHEMISTRY, MULTIDISCIPLINARY | 50 | 2.38% |
| CHEMISTRY, APPLIED | 49 | 2.33% |
| PHYSIOLOGY | 45 | 2.14% |
| MEDICINE, GENERAL & INTERNAL | 40 | 1.91% |
| ONCOLOGY | 39 | 1.86% |
| AGRICULTURE, DAIRY & ANIMAL SCIENCE | 39 | 1.86% |
| VETERINARY SCIENCES | 28 | 1.33% |
| BIOCHEMICAL RESEARCH METHODS | 27 | 1.29% |
| AGRICULTURE, MULTIDISCIPLINARY | 23 | 1.10% |
| TOXICOLOGY | 20 | 0.95% |
| ENVIRONMENTAL SCIENCES | 20 | 0.95% |
| GENETICS & HEREDITY | 18 | 0.86% |
| BIOLOGY | 17 | 0.81% |
| CARDIAC & CARDIOVASCULAR SYSTEMS | 16 | 0.76% |
| CHEMISTRY, ANALYTICAL | 13 | 0.62% |
| CHEMISTRY, MEDICINAL | 13 | 0.62% |
| NEUROSCIENCES | 11 | 0.52% |
| VIROLOGY | 10 | 0.48% |
| BIOPHYSICS | 8 | 0.38% |
| SURGERY | 5 | 0.24% |
| PATHOLOGY | 5 | 0.24% |
| PARASITOLOGY | 5 | 0.24% |
| INFECTIOUS DISEASES | 5 | 0.24% |
| CHEMISTRY, ORGANIC | 4 | 0.19% |
| ENGINEERING, CHEMICAL | 3 | 0.14% |
| INTEGRATIVE & COMPLEMENTARY MEDICINE | 3 | 0.14% |
| MEDICAL LABORATORY TECHNOLOGY | 3 | 0.14% |
| PUBLIC, ENVIRONMENTAL & OCCUPATIONAL HEALTH | 2 | 0.10% |
| MARINE & FRESHWATER BIOLOGY | 2 | 0.10% |
| HEMATOLOGY | 2 | 0.10% |
| FISHERIES | 2 | 0.10% |
| ECOLOGY | 2 | 0.10% |
| PHYSICS, APPLIED | 1 | 0.05% |
| MATERIALS SCIENCE, MULTIDISCIPLINARY | 1 | 0.05% |
| WATER RESOURCES | 1 | 0.05% |
| PERIPHERAL VASCULAR DISEASE | 1 | 0.05% |
| MEDICINE, LEGAL | 1 | 0.05% |
| NANOSCIENCE & NANOTECHNOLOGY | 1 | 0.05% |
| SUBSTANCE ABUSE | 1 | 0.05% |
| CRITICAL CARE MEDICINE | 1 | 0.05% |
| POLYMER SCIENCE | 1 | 0.05% |
| MICROSCOPY | 1 | 0.05% |
| EVOLUTIONARY BIOLOGY | 1 | 0.05% |
| DENTISTRY, ORAL SURGERY & MEDICINE | 1 | 0.05% |
